# Microbial Transformation of Hesperidin and Biological Evaluation

**DOI:** 10.1021/acsomega.3c05334

**Published:** 2023-11-01

**Authors:** Damla Kırcı, Fatih Demirci, Betül Demirci

**Affiliations:** † Department of Pharmacognosy, Faculty of Pharmacy, Selçuk University, Konya 42150, Türkiye; ‡ Department of Pharmacognosy, Faculty of Pharmacy, Anadolu University, Eskişehir 26470, Türkiye; § Faculty of Pharmacy, Eastern Mediterranean University, N. Cyprus, Via Mersin, Famagusta 99628, Türkiye

## Abstract

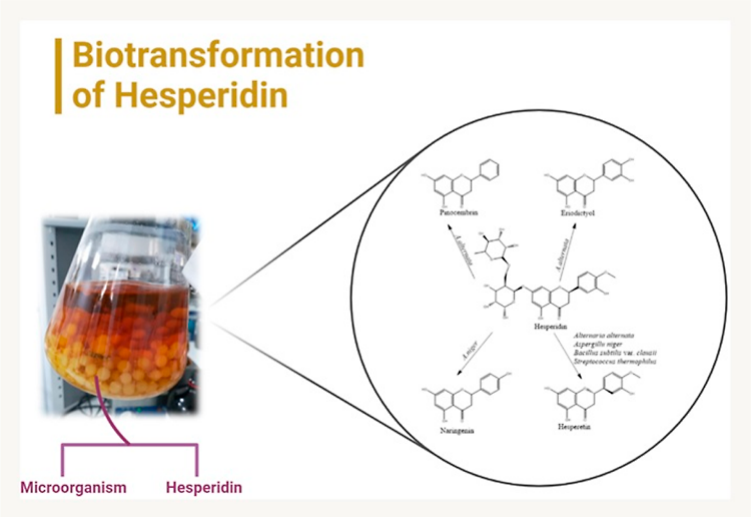

The main aim of the study was the biotransformation evaluation of hesperidin for functionalization by 25 different nonhuman pathogenic microorganisms. As a result, four metabolites were identified and characterized. The structure of pinocembrin and naringenin from the microbial transformation of hesperidin was determined initially with LC/MS–MS. The metabolites eriodictyol and hesperetin were isolated, and their molecular structure was determined by NMR and MS. Pinocembrin, eriodictyol, and naringenin were characterized as new hesperidin microbial transformation metabolites, to the best of our knowledge. In order to evaluate the bioactivity, in vitro 5-lipoxygenase (5-LOX) enzyme inhibition, antioxidant, antimicrobial, and acute toxicity evaluations using the brine shrimp assay of hesperidin and its metabolites were performed comparatively. According to antioxidant and anti-inflammatory activity results, hesperetin metabolite was more active than naringenin and hesperidin. The antimicrobial activity of hesperetin and naringenin against the human pathogenic *Staphylococcus aureus* strain was relatively higher when compared with the substrate hesperidin. In line with this result, biofilm activity of hesperetin and naringenin against *S. aureus* with combination studies using biofilm formation methods was carried out. The checkerboard combination method was utilized for biofilm layering, also for the first time in the present study. As an initial result, it was observed that hesperidin and naringenin exerted a synergistic activity with a fractional inhibitory concentration index (FICI) value of 1.063. Considering the bioactivity of hesperidin, hesperetin, and naringenin used as substrates are relatively nontoxic. The microbial and enzymatic biotransformation of natural products such as hesperetin and its new bioactive metabolites still have pharmacological potential, which needs further experimentation at the molecular level..

## 1 Introduction

Microbial transformation is the natural and biochemical transformation carried out in order to modify substances that enter the cell by various mechanisms, including detoxification, with the aid of enzymes under various conditions or to increase the efficiency of the compounds used due to their pharmacological effects, with the catalysis of the microorganism, cell, and isolated enzymes. Microbial transformation is a wide variety of biotechnological reactions, and one of its most important features is that it allows reactions which are challenging synthetically.^1^

Microbial and enzymatic transformation is an effective method for the structural change of natural compounds with known biological activity, including polyphenolic compounds, and for the biotransformation of new metabolites formed by this change.^2^

The well-known hesperidin (=hesperetin-7-*O*-rutinoside) is a flavone bearing rhamnose plus glucose and the hesperetin aglycone moiety. Hesperidin is also known to pose a relatively low water solubility and low bioavailability in oral intake due to the presence of a rutinoside part in its structure^3^ It is abundant in citrus fruits and is produced in the citrus industry. According to recent preclinical and clinical studies on the biological use of hesperidin as an active ingredient, it has antioxidant, anti-inflammatory, lipid-lowering, and insulin sensitivity properties and is effective in neurological disorders, psychiatric disorders, and cardiovascular diseases due to its effects on blood pressure; it is also used in food supplements due to its various biological activities.^4^

In our microbial transformation studies, hesperidin, as a natural product, was used as a substrate for microbial biotransformation, aiming for new bioactive derivatives. The biological effects of the purified, characterized new metabolites and hesperidin were compared using in vitro antioxidant, antimicrobial, as well as anti-inflammatory assays, complemented by the brine shrimp assay for initial toxicological data. For the biotransformation experimentation, 25 different nonpathogenic microorganisms were used; pathogens such as *Staphylococcus aureus*, however, were targeted for antimicrobial and antibiofilm formation assays, with various combinations aiming synergic combat.

## 2 Results and Discussion

### 2.1 Biotransformation of Hesperidin

In the microbial transformation trials of hesperidin, the presence of microbial transformation metabolites was inspected initially by thin-layer chromatography (TLC) analysis, comparing 25 microorganisms. Two of these strains were selected as bacteria (*Bacillus subtilis* var. clausii and *Streptococcus thermophilus*), one as yeast (*Sporobolomyces pararoseus*), and five as fungi selected from different species (*Alternaria alternata*, *Aspergillus niger*, *Rhizopus stolonifera*, *Fusarium solani,* and *Penicillium claviforme*) for further biotransformation scale up, all as nonhuman pathogens.

In line with the data obtained from the preliminary trials, preparative-scale studies were carried out for the isolation and purification of metabolites, and the structures of the four metabolites were determined using chromatographic and spectroscopic techniques.

#### 2.1.1 Metabolite 1

Metabolite purification from *Bacillus subtilis* var. clausii transformation extract was carried out by column chromatography. Metabolite 1 (M1) was characterized by using NMR and high-resolution mass spectrometry (HRMS) analyses. When ^1^H NMR spectra were compared with hesperidin spectra, there were new peaks in the range of 5.38–3.15 ppm in the hesperidin spectrum. These peaks indicate the presence of carbohydrate groups. At the same time, the peak observed at 1.07 ppm (3H, d, and *J* = 6.0 Hz) in the hesperidin spectrum was associated with the methyl peak in the carbohydrate group. Hesperidin with two hydroxy groups at 12.01 and 9.08 ppm is present as a singlet in the spectrum. However, the new M1 metabolite showed three hydroxy groups in its structure. In the metabolite spectrum, it was observed as a singlet at 12.18, 9.60, and 7.73 ppm.

**Figure 1:**
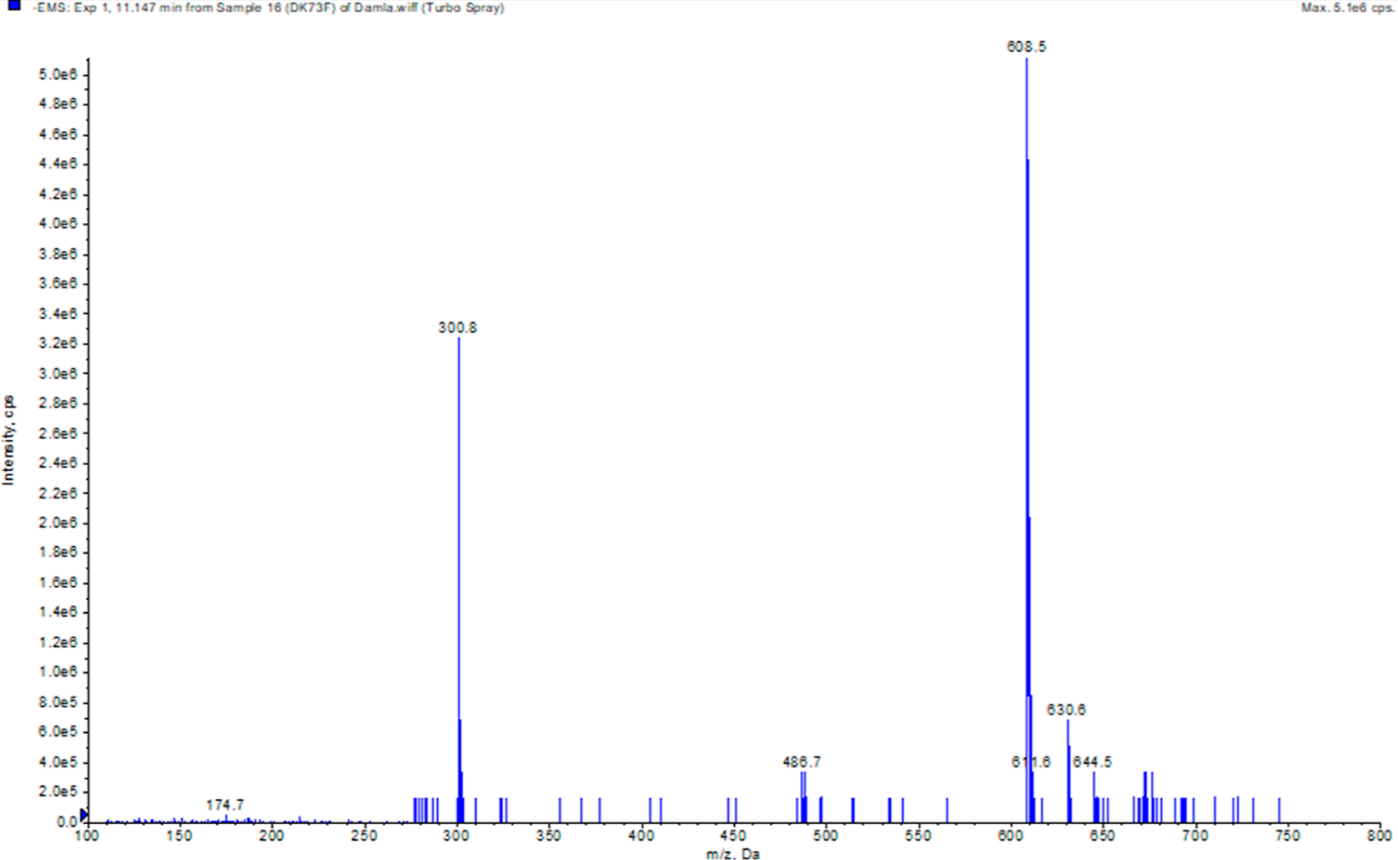
Mass spectrum of hesperidin.

**Figure 2:**
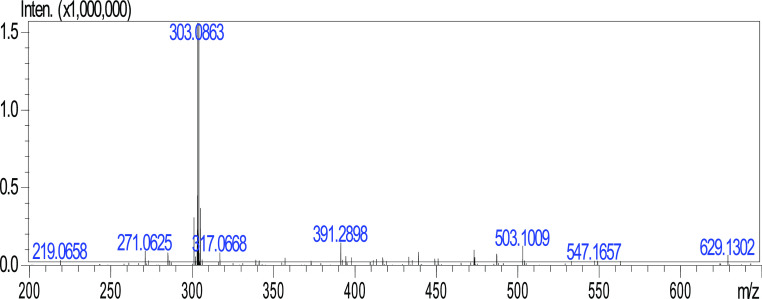
HRMS spectrum of hesperetin.

In the ^13^C NMR spectrum of Hesperidin, peaks in the range of 66–130 ppm were observed associated with the carbohydrate group, and the methyl peak was observed at 1.83 ppm. While the methoxy peak was observed at 56.33 ppm in the M1 metabolite, the same peak was observed at 55.66 ppm in hesperidin. According to the NMR and mass spectrum values obtained, it was concluded that the M1 metabolite was hesperetin, which is the known aglycone form of hesperidin, as illustrated in Figures 1 and 2.

**Figure 3:**
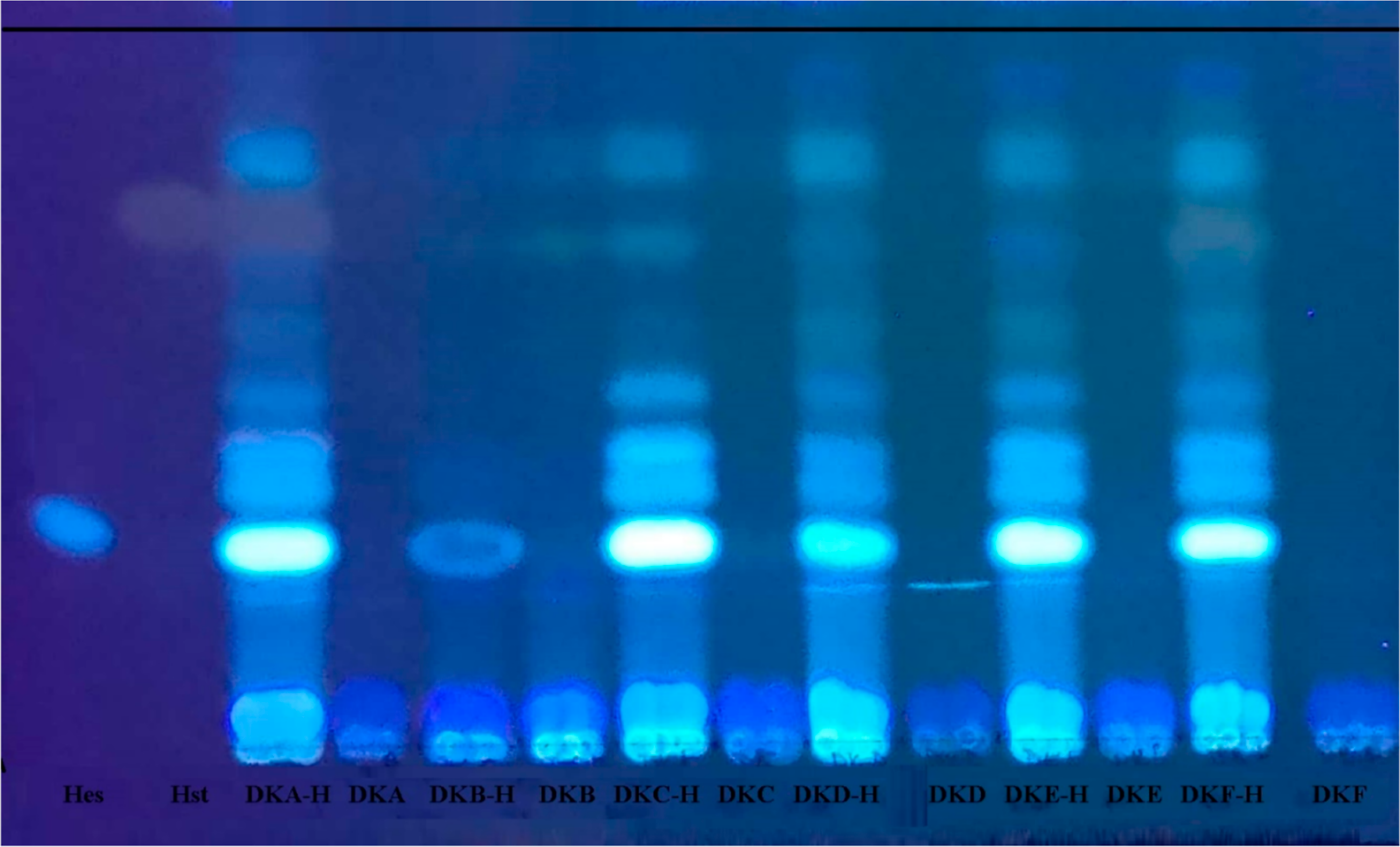
Microbial transformation of hesperidin by bacteria (TLC image at 365 nm). **H**: hesperidin, **Hst**: hesperetin, **DKA-H**: *Bacillus subtilis* var. clausii transformation extract; **DKA-blank**: *Bacillus subtilis* var. clausii blank extract; **DKB-H**: *Bacillus coagulans* transformation extract; **DKB-blank**: *B. coagulans* blank extract; **DKC-H**: *B. subtilis* var. notto transformation extract; **DKC-blank:***B. subtilis* var. notto blank extract; **DKD-H**: *Lactobacillus fermentum* transformation extract; **DKD-blank**: *L. fermentum* blank extract; **DKE-H**: *Lactobacillus rhomnosus* transformation extract; **DKE-blank**: *L. rhomnosus* blank extract; **DKF-H**: *S. thermophilus* transformation extract; **DKF-blank**: *S. thermophilus* blank extract.

TLC plate analyses of the microbial transformation study performed with six bacteria within the scope of the study are shown in Figure 3 at 365 nm. The metabolite M1, which is hesperetin by *Bacillus subtilis* var. clausii, was purified and isolated by the prepTLC method. The isolated metabolite was also observed in the *S. thermophilus* biotransformation extracts.

Hesperetin (3′,5,7-trihydroxy-4′-methoxyflavanone) is a well-known flavonoid with a flavanone structure. It is found in citrus fruits (Rutaceae); however, it is also in the peel of *Citrus sinensis* L. and orange juice.^5^ Similar to most flavonoid compounds with a hydrophobic structure, hesperetin is known to have poor water solubility and low absorption in the gastrointestinal tract.^6^

In addition, it is known that hesperetin is a vasoprotective agent that is highly effective in the treatment of hemorrhoids and in preventing postoperative thromboembolism. Hesperetin has promising antioxidant (acting mainly as a scavenger of free radicals), estrogenic, anti-inflammatory, anticancer, antidiabetic, antiatherogenic, and cardioprotective effects. Hesperetin can be obtained from the hydrolysis of hesperidin by the action of intestinal bacteria.^5,6^

According to recent biosynthesis data, hesperetin synthesis in plants and microorganisms is carried out by the enzyme eriodictyol 4′-*O*-methyltransferase. Hesperetin is glycosylated at the C-7 position of 7-*O*-glucosyltransferase; glucose is bound, and hesperetin-7-*O*-glucose is formed.^3,7^ This biotransformation was observed by *Pichia kluyverii* human faeces flora isolated from *Aspergillus* and *Cunninghamella* genera, *Rhizopus stolonifer*, *Gliocladium roseum*, *Paecilomyces variotii*, and *Streptomyces griseus* microbiota.^8−15^

#### 2.1.2 Metabolite 2

A manual column chromatography study was performed with the transformation extract obtained from the preparative scale with *A. alternata*. As a result of this study, the structures of three metabolites detected in the transformation extract were examined by spectroscopic methods.

When the ^1^H NMR spectrum of metabolite 2 (M2) was compared with the hesperidin spectrum, there were peaks belonging to the carbohydrate group in the range of 5.38–3.15 ppm, a doublet peak 1.07 (3H, d, *J* = 6.0 Hz) of the methyl as well.

**Figure 4:**
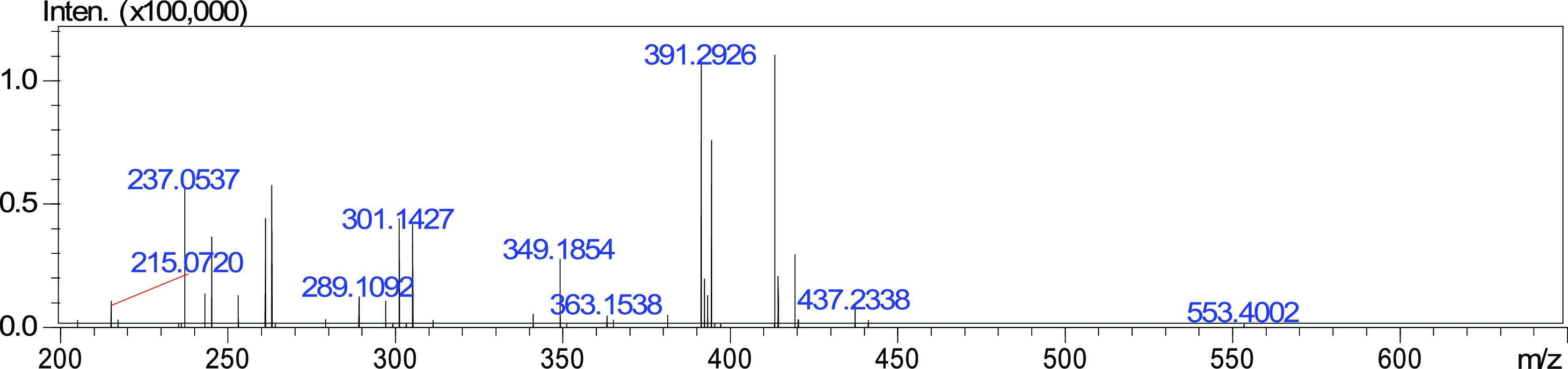
HRMS spectrum of eriodictyol.

The M2 metabolite contained four hydroxy groups in its structure and showed a singlet at 12.68, 9.45, 8.98, and 8.54 ppm, respectively. Additionally, 8.98 ppm in the spectrum of the M2 metabolite characteristically indicated the presence of a hydroxy group at C-4. In the hesperidin ^13^C NMR spectrum, the peaks in the range of 66–130 ppm belonging to the carbohydrate moiety, and the peak signal of the methyl was observed here at 1.83 ppm. While a methoxy peak was observed at 55.66 ppm in hesperidin; however, the peak was absent in M2. HRMS analysis of M2 revealed [M – H]^+^ 288.2916, as shown in Figure 4. The metabolite was confirmed as eriodictyol with the interpretation of NMR and HRMS analysis results and with the support of the data in the literature.^16,17^

As is known, eriodictyol (=3′,4′,5,7-tetrahydroxyflavanone) is a flavonoid which is present in the Citrus genus and other vegetables. Eriodictyol showed pharmacologically antioxidant, antidiabetic, anti-inflammatory, anticancer, neuroprotective, and hepatoprotective effects.^18^ According to the biosynthesis pathway in plants and microorganisms, the synthesis of eriodictyol shares the same pathway as hesperidin. In the plant biosynthesis pathway, first eriodictyol and then hesperidin transformation was observed.^3^

#### 2.1.3 Metabolite 3

A manual column chromatography study was performed with the transformation extract obtained from the preparative scale with *A. alternata*. As a result of this study, the structures of two metabolites detected in the transformation extract were examined by spectroscopic methods. One of the metabolites was eriodictyol (M2). While hesperetin was observed as a metabolite in the 40–50% *n*-hexane/ethyl acetate fraction obtained from column chromatography, the new metabolite 3 (M3) was also observed in the fraction. The mass spectrum for M3 was similar to that for M2 [M – H]^−^ 254.9.

**Figure 5:**
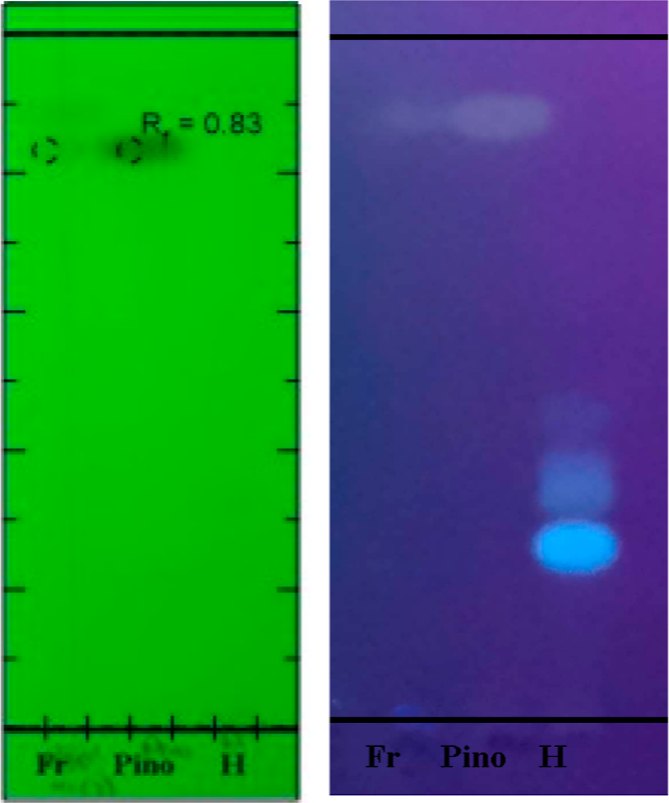
Column fraction (Fr), pinocembrin (Pino), and hesperidin (H) TLC at 254 and 365 nm.

**Figure 6:**
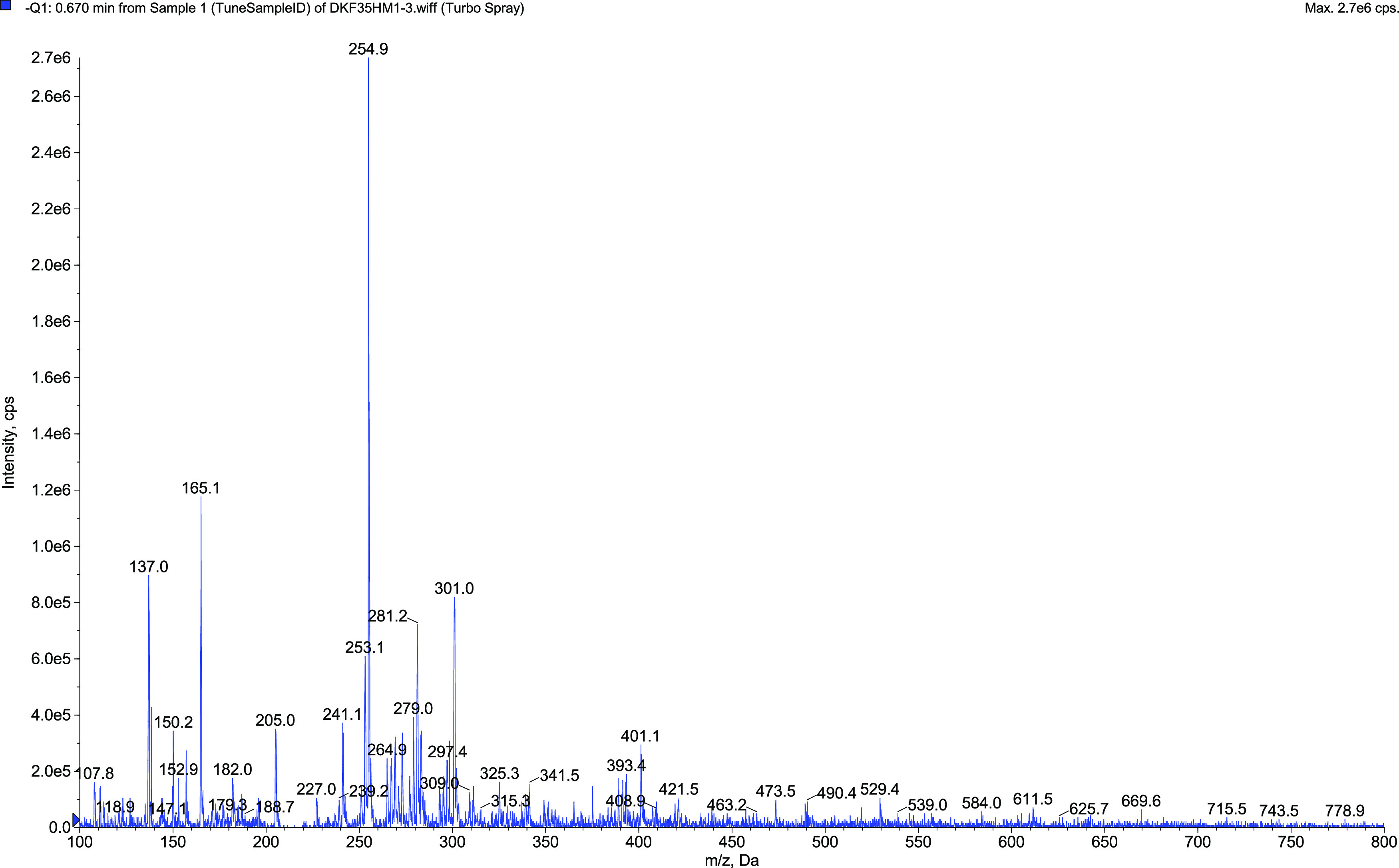
Mass spectrum of pinocembrin.

The fraction obtained from the transformation extract with the standard sample of pinocembrin was analyzed by TLC (Figure 5). According to the TLC and LC–MS/MS analysis results of the fraction, it was confirmed that the new metabolite was pinocembrin, as shown in Figure 6. The cleavage of the carbohydrate groups of hesperidin occurs due to the cleavage of the hydroxy groups at the 3′ position as well as the methoxy groups at the 4′ position in ring B.

Pinocembrin (5,7-dihydroxyflavanone) is also a known flavonoid, which is mostly found as a propolis component. It was previously isolated from the Peperomia Ruiz & Pav. and Piper L. genera and the Asteraceae family. Pinocembrin has a low molecular weight and lipophilicity, allowing it to pass easily through the blood–brain barrier. The pharmacological effect of this compound was reported on its anti-inflammatory, antioxidant, antibacterial, and neuroprotective activities.^19,20^

It is known that in plants, pinocembrin is synthesized from phenylalanine through phenylalanine ammonia lyase, 4-coumarate CoA ligase, and chalcone synthase, respectively. Accordingly, *Saccharomyces cerevisiae* and *Escherichia coli* are successful catalysts utilizing glucose by recombinant gene transfer for large-scale production.^19,21^ This data supports the transformation of pinocembrin and eriodictyol metabolites.^22^

According to the previous work^22^ of Nikolic and van Breemen (2004), transformation metabolites obtained from pinocembrin were investigated using rat liver microsomes. Transformation of pinocembrin to naringenin via the 4′-hydroxylation pathway was observed. Eriodictyol transformation was observed by adding hydroxy to the 3′ position of the B ring of naringenin. Two metabolites observed in this study were obtained from different pathways in the shikimic acid pathway. This study showed that the metabolites were obtained with liver microsomes and the formation of naringenin, which was obtained as an intermediate step of the two metabolites. Literately, pinocembrin and eriodictyol were synthesized in rat liver microsomes and the plant. However, they were obtained by *A. alternata* for the first time in the present study.

#### 2.1.4 Metabolite 4

Two new metabolites were observed in the TLC plate analyses from the biotransformation results using *A. niger*. The mass spectra were confirmed by a comparison of standard samples of hesperetin (M1) and naringenin (M4), respectively.

**Figure 7:**
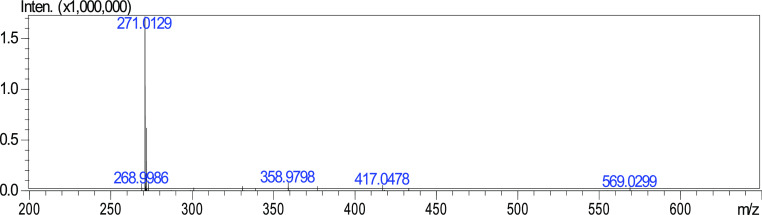
HRMS spectrum of naringenin.

The biotransformation was repeated on the 4th, 8th, and 12th days for LC–MS/MS and HRMS analyses (Figure 7). The bioformation of naringenin on the 4th day was less compared to hesperetin amounts in the extracts from three different intervals, which supports the findings.

Hesperetin metabolite was formed after the carbohydrate moieties in hesperidin were cleaved and transformed into aglycons. Then, the metabolite naringenin was obtained by the removal of the methyl group at the 4′ position in the B-ring and the dehydroxylation reactions at the 3′ position, respectively.

Naringenin (4′,5,7-trihydroxyflavan-4-one) is a flavone which is commonly found in some *Citrus* species, tomatoes, and the fruit of the *Ficus carica* species. Naringenin pharmacologically has antioxidant, antihypertensive, anti-inflammatory, antidiabetic, hypoglycemic, hypolipidemic, cardioprotective, hepatoprotective, and protective effects against obesity.^5,23^

**Scheme 1:**
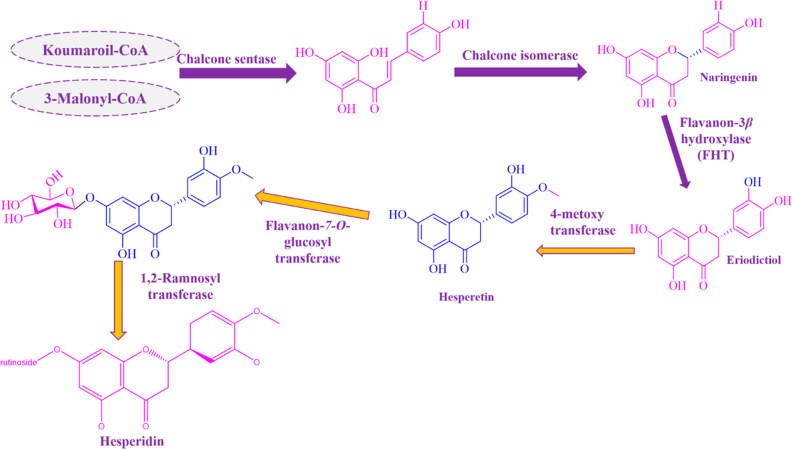
Biosynthesis of Hesperidin in the Plant^3^

Naringin is moderately soluble in water and highly soluble in organic solvents, such as alcohol. Intestinal microflora converts naringin to the aglycone naringenin in the intestine and provides absorption from the intestine.^23^ Naringenin is transformed from chalcone by an isomerase in the biosynthesis pathway observed in plants and microorganisms, as illustrated in Scheme 1.^3^ According to this pathway, naringenin, followed by hesperetin and hesperidin transformation, can be observed, respectively.

**Scheme 2:**
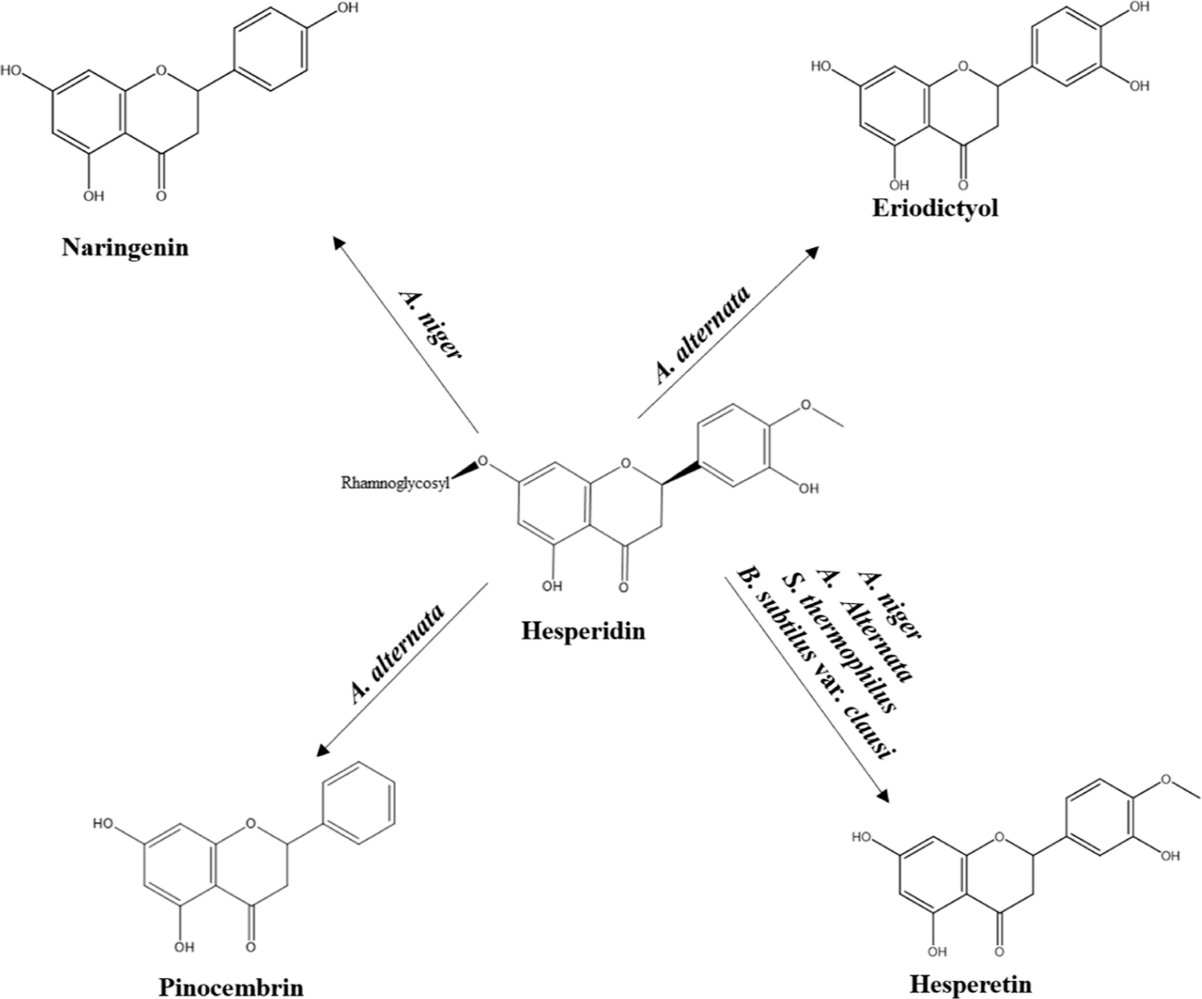
Microbial Transformation of Hesperidin

In this study, the sequential transformation in the biosynthesis pathway occurs in the opposite direction. The metabolites of the structures, which were determined by microbial transformation studies of hesperidin, are shown in Scheme 2.

### 2.2 Biological Activities

In vitro antioxidant, 5-LOX enzyme inhibition, antimicrobial, and acute toxicity activities were evaluated comparatively using hesperidin, hesperetin, and naringenin.

#### 2.2.1 Antioxidant Activity

Two different methods, DPPH^·^ radical scavenging and ABTS^·+^, were used for antioxidant activity studies. DPPH^–^ radical scavenging and ABTS^–+^ results based on hesperidin, hesperetin, and naringenin concentrations are listed in Tables 1 and 2, respectively. In the DPPH^·^ radical scavenging assay, ascorbic acid was used as a positive control, the IC_50_ value was calculated as 15.495 ± 0.6 μg/mL, and the % inhibition of ascorbic acid (25 μg/mL concentration) was 89.69 ± 3.79%. Hesperetin metabolite, one of the hesperidin metabolites, showed antioxidant activity higher than that of naringenin metabolite. According to the results of this study, the activities of metabolites were relatively less compared to those of the substrate.

**Table 1:** DPPH^·^ Radical Scavenging Activity (% Inhibition) of Hesperidin and Its Metabolites

	Concentration				
	25 μg/mL	50 μg/mL	100 μg/mL	200 μg/mL	*p*
hesperidin	11.796 ± 1.09^a^	15.782 ± 3.23^a^	25.639 ± 3.62^a^	31.437 ± 4.41^a^	0.017
hesperetin	14.042 ± 0.77^a^	14.332 ± 1.26^a^	17.956 ± 1.53^a^	21.242 ± 4.94^a^	0.067
naringenin	13.511 ± 1.23^a^	15.637 ± 2.8^a^	18.608 ± 1.81^a^	18.198 ± 2.34^a^	0.067
ascorbic acid	89.69 ± 3.79^a^	93.91 ± 0.13^a^	94.13 ± 0.15^a^	95.68 ± 1.95^a^	<0.001

a There is no difference between groups with the same letter for each measurement value. The data was analyzed by ANOVA Tamhane.

**Table 2:** ABTS^·+^ Antioxidant Evaluation (% Inhibition) of Hesperidin and Its Metabolites

	Concentration				
	250 μg/mL	500 μg/mL	1 mg/mL	2 mg/mL	*p*
hesperidin	5.8 ± 1.80^c^	13.9 ± 0.93^b^	22.6 ± 1.10^a^	27.9 ± 2.82^a^	<0.001
hesperetin	13.6 ± 1.01^c^	19.8 ± 0.13^b^	22.8 ± 3.81^a^^b^^c^	35.1 ± 1.53^a^	0.001
naringenin	9.9 ± 1.12^a^	10.1 ± 0.82^a^	12.8 ± 0.82^a^	16.4 ± 1.85^a^	0.016
trolox	70.50 ± 4.51^a^	82.58 ± 0.23^a^	82.79 ± 0.40^a^	83.09 ± 0.42^a^	<0.001

a ^,^

b ^,^

c ^,^

There is no difference between groups with the same letter for each measurement value. The data was analyzed by ANOVA Tukey HSD.

The positive control of ABTS^·+^ antioxidant activity was Trolox (IC_50_: 34.703 ± 1.7 μg/mL), and the percentage inhibition of 250 μg/mL concentration of Trolox was shown as 70.50 ± 4.51%. An activity too low to calculate the IC_50_ value was observed, although the activity of the hesperetin metabolite, one of the hesperidin transformation metabolites, was higher than that of naringenin.

As a result of the studies with hesperidin, naringenin, and hesperetin, while hesperetin has the highest effect according to different antioxidant activity methods, hesperidin also showed a higher inhibition compared to naringenin in terms of antioxidant activity.^24^

#### 2.2.2 Anti-inflammatory Activity

**Figure 8:**
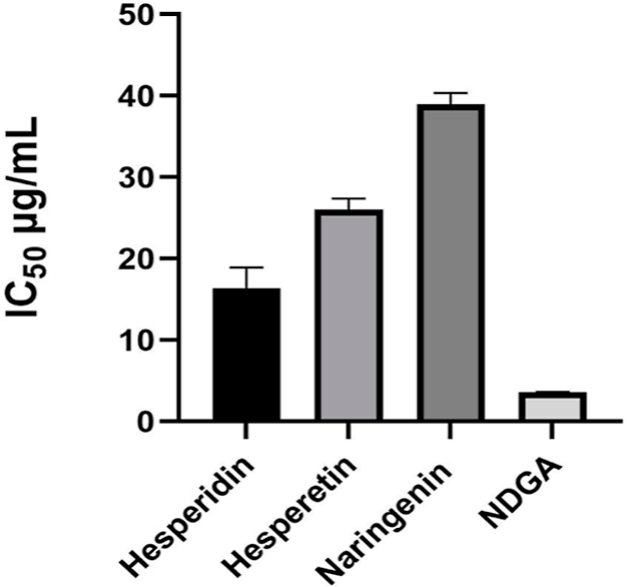
IC_50_ values of hesperidin and its metabolites.

Soy 5-LOX enzyme was used in the anti-inflammatory activity study. Within the scope of this activity study, NDGA was used as the positive control, and the IC_50_ value was calculated as 3.63 ± 0.29 μg/mL. In contrast, the hesperidin IC_50_ value was calculated as 17.21 ± 2.10 μg/mL (Figure 8). Anti-inflammatory activity results are listed in Table 3. The hesperetin metabolite and hesperidin were showed 72.88 ± 2.48% and 61.51 ± 4.01% inhibition at 40 μg/mL concentrations, respectively. These results indicate the production of a more active metabolite from hesperidin (substrate). Furthermore, according to both antioxidant and anti-inflammatory activity results, hesperetin was relatively more active than the naringenin metabolite.

**Table 3:** Anti-inflammatory Activity (% Inhibition) of Hesperidin and Its Metabolites by 5-LOX Evaluation

	concentration			
	10 μg/mL	20 μg/mL	40 μg/mL	*p*
hesperidin	43.58 ± 4.53^b^	51.58 ± 3.34^a^^b^	61.51 ± 4.01^a^	0.004
hesperetin	6.25 ± 3.87^c^	36.01 ± 4.85^b^	72.88 ± 2.48^a^	<0.001
naringenin	35.32 ± 6.55^b^	41.27 ± 1.24^a^^b^	51.39 ± 4.14^a^	0.013
NDGA	54.97 ± 0.10^b^	84.80 ± 0.17^a^^b^	95.12 ± 0.14^a^	<0.001

a ^,^

b ^,^

c ^,^

There is no difference between groups with the same letter for each measurement value. The data was analyzed by ANOVA Tukey HSD.

To the best of our knowledge, the anti-inflammatory activity study of hesperidin and hesperetin using the 5-LOX enzyme was performed for the first time. While IC_50_ values were calculated for both compounds as a result of other in vitro activity studies, the activity result of hesperidin was relatively greater compared to hesperetin. The results obtained within this study were consistent with the literature.^6,24^

#### 2.2.3 Antimicrobial Activity

Within the present antimicrobial activity studies, ten microorganisms were evaluated. For the selection, strains with the biofilm layer formation ability were preferred. In addition, yeasts present in the human intestinal flora, which positively affect human health, were also selected. Six bacteria (*E. coli* NRRL B-3008, *S. aureus* ATCC 6538, *Salmonella typhimurium* ATCC 13311, *Bacillus subtilis* NRRL B-4378, *Bacillus fragilis*, and Bacillus licheniformis), two yeasts (Saccharomyces boulardii REFLOR and *S. cerevisiae* ATCC 9763), and two fungi (*Candida albicans* ATCC 10231 and *Aspergillus nidulans* Abraham) were used, where the antimicrobial activity results are listed comparatively in Table 4.

**Table 4:** Antimicrobial Activity of Hesperidin and Its Metabolites (μg/mL)

	hesperidin	hesperetin	naringenin	B1	B2
*Escherichia coli*	>500	>500	>500	<10	5
*Staphylococcus aureus*	125	**62.5**	**31.25**	<10	10
*Salmonella typhimurium*	>500	500	500	<10	10
*Bacillus subtilis*	>500	500	500	<10	5
*Bacillus fragilis*	>500	500	500	40	10
*Bacillus licheniformis*	>500	500	500	40	20
*Candida albicans*	<15.6	<15.6	<15.6	4^a^	2^b^
*Saccharomyces boulardii*	>500	62.5	125	1^a^	0.5^b^
*S. cerevisiae*	>500	125	125	2^a^	2^b^
*Aspergillus nidulans*	<15.6	<15.6	31.25	2^a^	0.12^b^

**B1:** amoxicillin; **B2:** chloramphenicol:

a Ketoconazole (μg/mL).

b Amphotericin B (μg/mL).

Hesperidin and its metabolites showed relatively high antifungal activity and inhibitory effects against the tested fungi. Test below 15.6 μg/mL concentration could not be performed due to the lack of a sufficient sample for the activity study. However, naringenin showed strong inhibitory activity against *S. aureus* strains compared to hesperidin. This result indicates that more effective metabolites with potential against skin pathogens can be achieved.

After the minimum inhibition concentration (MIC) study, minimum bactericidal concentration (MBC) and minimum fungicidal concentration (MFC) studies were performed. MBC and MFC give information about the concentration at which the compounds have an inhibitory or lethal effect on microorganisms.

According to MBC results, only the naringenin metabolite had a bactericidal effect against the *S. aureus* strain at a concentration of 500 μg/mL. Flavonoids have a higher antibacterial effect against the *S. aureus* strain due to the hydroxy groups connected to the fifth and seventh positions. The MFC of the hesperetin metabolite against *A. nidulans* was found to be 62.5 μg/mL.^25^ The literature includes studies on hesperidin, hesperetin, and naringenin.^26−28^

#### 2.2.4 Biofilm Activity

Biofilm refers to the community formed by microorganisms adhering to each other and to a living or nonliving solid surface. Many sinusitis agents, such as *S. pneumoniae,H. influenza*, *S. aureus,* and *Pseudomonas aeruginosa,* cause disease by forming biofilms in the pathogenic sinuses and their removal from the environment is more difficult than single living microorganisms. Therefore, they are resistance factors.^29^

In this context, a biofilm study was carried out with *S. aureus* (ATCC 6538) and *P. aeruginosa* (ATCC 27853). Hesperetin and naringenin compounds with strong inhibitory activities were selected for the study. In addition, hesperidin was also included in the study since it is a metabolite of hesperidin. MIC and MBIC values of the tested three compounds are listed in Table 5.

**Table 5:** MIC and MBIC Values of Hesperidin and Its Metabolites (μg/mL)

microorganisms	MIC/MBIC (μg/mL)	hesperidin	hesperetin	naringenin
*Staphylococcus aureus*	MIC	125	62.5	31.25
	MBIC	500	250	250
*Pseudomonas aeruginosa*	MIC	500	500	500
	MBIC	>500	>500	>500

As a result of the biofilm and antibacterial activity study, hesperetin and naringenin were relatively more effective compared to hesperidin. According to the literature, a biofilm study of naringenin with *Vibrio harveyi* and *E. coli* strains was reported.^30^

In another study, it was determined that naringenin decreased biofilm formation by reducing fatty acid secretion with antibacterial activity on *S. aureus* MRSA mutants. In addition, high synergistic activity was observed with the oxacillin combination, which is used in treatment for both antibacterial activity and biofilm inhibition.^31^

Another strain in which naringenin was studied was *Streptococcus mutans*. The biofilm formed using this strain suppresses the second (bacterial adhesion) and third stages (biofilm maturation). As a result of this study, it is predicted that naringenin can be used as a safe anticaries agent at appropriate concentrations in dental clinics.^32^

In the biofilm study performed with hesperidin and hesperetin compounds, hesperetin was relatively more active against the *S. aureus* RN4220 and *S. aureus* SA1199B strains, respectively. Different compounds in glycoside and aglycone forms were compared, and it was concluded that flavonoids in aglycone structures were more effective than glycoside forms.^33^

#### 2.2.5 Synergistic Antibacterial Activity

A combination study was carried out since a significant result was observed against *S. aureus* in biofilm layering. Therefore, after the biofilm structure of *S. aureus* (ATCC 6538) was formed with naringenin and hesperetin, the study was carried out with the concentrations by the checkerboard method. As a result of the study, the FICI value was calculated according to the formula “FICI = FIC X + FIC Y”. [X for hesperetin (FICX: 0.25) and Y for naringenin (FICY: 0.0625)].

It was observed that the fractional inhibition was ∑FICI = 0.3125, corresponding to the experimental study in the range of FICI ≤ 0.5 for the “synergistic effect”. Thus, as a result, hesperetin and naringenin showed a synergistic effect on the biofilm plate.

In the literature, a synergistic activity study of naringenin and hesperetin against the *S. aureus* ATCC 12598 strain was conducted, and the FICI value was 1.063, justified as an “independent effect”. While it has an independent effect as a synergistic effect, it was observed that it showed a synergistic effect on the biofilm layering.^34^

For bacteria grown in benzylactam antibiotics, flavonoids showed no effect on beta-lactamase enzymes and PBP-2 levels. An electron microscopy study was carried out to analyze the mechanism of action. As a result, abnormal morphology was observed in bacteria treated with flavonoids at lower inhibitory concentrations.^35^

#### 2.2.6 Cytotoxicity

*Artemia salina* (Brine Shrimp) are zooplanktonic crustaceans that can survive in different living conditions, such as swamps and lakes, due to their ability to adapt well to extreme salinity. Since these creatures are filter feeders, they are used in toxicity experiments because they show extreme sensitivity to toxic substances. These studies with *Artemia* eggs are accepted as an in vivo animal alternative model.^36^ The positive control was emetine hydrochloride, where the lethal percentage results were at 62.50 μg/mL concentration of substrate and metabolites are listed in Table 6.

**Table 6:** Acute Toxicity of Hesperidin and Its Metabolites by Brine Shrimp Assay

	lethal percentage (%)
hesperidin	a
hesperetin	8.01 ± 0.45
naringenin	4.55 ± 6.43
emetine hydrochloride	100 ± 0.0

a Not effected.

Toxicity is divided into four classes according to LC_50_ values in the brine shrimp experiment. These toxicity ratings are strongly toxic (LC_50_ value: <100 μg/mL), moderately toxic (LC_50_ value: 100–500 μg/mL), weakly toxic (LC_50_ value: 500–1000 μg/mL), and nontoxic (LC_50_ value: >1000 μg/mL).^36^ Considering the calculations, hesperidin, hesperetin, and naringenin used as substrates were nontoxic. Previous toxicity studies of hesperidin, hesperetin, and naringenin also supported the results. Although there are previous studies of hesperidin toxicity using in vivo animal modeling and other in vivo animal alternative models, to the best of our knowledge, there is no study with the brine shrimp method where a correlation with the results in the literature exists.^37−39^

Overall, within the present study, microbial transformation studies were carried out with 25 different microorganisms based on hesperidin. They were found in *Citrus* fruit as vegetable food waste and used as a food supplement due to their strong biological activities. In this study, the transformation of compounds that are less common in nature compared with hesperidin was carried out. Pinocembrin, eriodyctiol, and naringenin as flavonoids were obtained by microbial transformation of hesperidin for the first time. This study was in accordance with and supported by the biosynthesis pathway in plants. In addition, the transformation of hesperidin to hesperetin with *A. alternata* and *S. thermophilus* was observed for the first time in the present study.

It was observed that hesperidin metabolites were more active than hesperidin in biological activity evaluations. The synergistic effect of hesperetin and naringenin metabolites against the biofilm was detected for the first time. Due to the observed synergistic effect of these metabolites, it was shown that they could be an effective area of use against the biofilm layer in combination. The biofilm checkerboard method was studied for the first time. The reproducible and self-consistent results of the study show that the method is promising. Hesperidin and its metabolites are not toxic according to the acute toxicity assessment ranges at the same concentrations of the metabolites whose substrate and other activity studies were performed with the Brine Shrimp method, which is one of the in vivo animal alternative methods.

## 3 Conclusions

The promising microbial transformations can be extended to enzymatically targeted transformations to obtain potential new bioactive metabolites for industrial utilization. New biotechnologically produced natural products with new biological evaluations can be extended to molecular or in vivo studies with promising potential new applications. However, more detailed toxicological studies and pharmacological evaluations are still needed.

## 4 Materials and Methods

### 4.1 Substrate and Microorganisms

Hesperidin was purchased from Sigma-Aldrich, Germany (>95%), also, analytical standards were of high purity from the same company or Merck (Germany), if not otherwise stated. The following microorganisms were utilized in microbial transformation reactions: *Bacillus subtilis* var. clausii (ATCC 9799), *B. coagulans* (SNZ 1969), *B. subtilis* var. notto (BN), *L. fermentum* (CECT-5716), *L. rhomnosus* (GG), *S. thermophilus* (TH-Ç), *R. stolonifer* (MF461023), *Aspergillus parasiticus* (NRRL 2999), *A. niger* (NRRL 326), *A. niger* (ATCC 10549), *A. niger* (NRRL 567), *A. alliaceus* (NRRL 317), *A. flavus* (ATCC 9807), *A. nidulans* (Abraham), *A. terreus* var. africanus (isolate), *P. claviforme* (MR 376), *P. valentinum* (isolate), *P. chrysogenum* (NRRL 792), *F. solani* (ATCC 1284), *F. culmorum* (isolate), *S. cerevisiae* (ATCC 9763), *S. pararoseus* (ATCC 11385), *A. alternata* (NRRL 20593), *Neurospora crassa* (isolate), and *Phanerachaetechrysosporium* (E 446).

### 4.2 Conditions of Cultivation and Transformation

The culture was kept and precultured on potato dextrose agar (PDA) slants at 5 and 25 °C, respectively. α-Medium was used in a microbial transformation assay with fungi prepared, whereas Mueller Hinton Broth was used for the yeast and bacteria. Microorganisms were grown aerobically at 150 rpm on a shaker incubator at 24 °C. After 2 days of growth, hesperidin was added, and the fermentation was maintained for another 12 days.^40^ Samples were collected from the fermentation medium for 12 days. The samples were extracted with ethyl acetate three times. After that, the extracts were dried over Na_2_SO_4_ (Merck) and the ethyl acetate solvent evaporated. Next, the extracts were given residue chromatography on silica with increasing concentrations of ethyl acetate in *n*-hexane.

### 4.3 Isolation of Metabolites

The residue was purified by using column chromatography using a glass column (3 cm wide and 60 cm long) and an MPLC system. Approximately powdery silica gel was used as the stationary phase, and glass tubes (1.5 cm wide and 10 cm long) were used to collect the fraction. Increasing concentrations of ethyl acetate in *n*-hexane were used as an eluent system.

### 4.4 Characterization of Transformation Metabolites

#### 4.4.1 LC–MS/MS Analysis

LC–MS/MS (Shimadzu, Kyoto, Japan) analysis of the extracts of microbial transformation assessed using a previously described process.^41^ LC-ESI-MS/MS data were collected and processed with Analyst 1.6 software. Separations were performed on a 150 × 3 mm i.d. RP-C18 analytical column (Supelco, Bellefonte, PA, USA).

Elution was carried out using a linear gradient of the solvent mixtures MeOH/H_2_O/CH_3_COOH (10:89:1, v/v/v) (solvent A) and MeOH/H_2_O/CH_3_COOH (89:10:1, v/v/v) (solvent B). The composition of B was increased from 10 to 100% in 20 min and then returned to the initial condition in 2 min.

#### 4.4.2 NMR Analysis

NMR spectra of hesperidin and the metabolites were recorded using acetone-*d*_6_ and DMSO-*d*_6_ as solvents, with tetramethylsilane (TMS) as an internal standard, on a Bruker spectrometer (300 MHz for ^1^H NMR and 75 MHz for ^13^C NMR). Coupling constants (*J*) were given as Hertz.

Hesperidin: ^1^H NMR (400 MHz, DMSO-*d*_6_): 1.08 (3H, d, *J* = 6.0 Hz, Me-5‴), 2.76 (1H, dt, *J* = 16.8 Hz, *J*o = 4.7 Hz, H-3α), 3.13 (1H, m, H-5‴), 3.21 (1H, m, H-4‴), 3.25 (1H, dd, *J* = 8.9 Hz, *J*o = 4.4 Hz, H-3β), 3.39 (1H, t, *J* = 4.3 Hz, H-3‴), 3.42 (1H, t, *J* = 4.3 Hz, H-2‴), 3.53 (1H, m, H-5′‘), 3.62 (1H, t, *J* = 4.1 Hz, H-3′‘), 3.76 (1H, s, –OMe-4′)4.46 (1H, d, *J* = 6.0 Hz, H-2″), 4.51 (1H, d, *J* = 1.6 Hz, H-4″), 4.59 (1H, d, *J* = 4.5 Hz, H-6″α), 4.67 (1H, d, *J* = 5.5 Hz, H-6″β), 4.97 (1H, d, *J* = 5.5 Hz, H-1‴), 5.38 (1H, d, *J* = 4.9 Hz, H-1″), 5.51 (1H, dd, *J* = 12.2 Hz, *Jo* = 3.3 Hz, H-2), 6.12 (2H, dd, *J* = 6.4 Hz, *J*o = 2.2 Hz, H-2′-H6′), 6.90 (1H, d, *J* = 8.1, H-5′), 6.93 (2H, dd, *J* = 5.2 Hz, *J*o = 3.2 Hz, 6H–8H), 9.08 (1H, s, OH-3′), 12.01 (1H, s, OH-5); ^13^C NMR (100 MHz, DMSO-*d*_6_): 17.83 (Me–C5‴), 42.04 (C-3), 55.68 (OMe-C4′), 66.03(C-6″), 68.31 (C-2‴), 69.58 (C-4″), 70.26 (C-3‴), 70.69 (C-5‴), 72.06 (C-3′‘), 72.98 (C-2″), 75.51 (C-4‴), 76.26 (C-5′‘), 78.37 (C-2), 96.37 (C-8), 99.43 (C-6), 100.60 (C-1‴), 103.31 (C-10), 112.02 (C-3′), 114.14 (C-6′), 117.94 (C-2′), 130.89 (C-1′), 130.96 (C-1″), 146.45 (C-5′), 147.95 (C-4′), 162.49 (C-9), 163.03 (C-5), 165.13 (C-7), 197.02 (C-4);^42^ KS (ESI) (*m*/*z*) [M + H]^−^: 608.6.

Metabolite 1 (M1) (Hesperetin) (12.5 mg) was isolated by prepITK with 10–20% *n*-hexane:ethyl acetate fraction obtained from the established column. The metabolite M1, obtained more polar than hesperidin, was pinkish at 365 nm and showed a black stain at 254 nm on the TLC plate.

^1^H NMR (400 MHz, DMSO-*d*_6_): 2.76 (1H, dd, *J* = 3.1 Hz, *Jo* = 0.8 Hz, H-3α), 3.18 (1H dd, *J* = 4.5 Hz, *Jo* = 1.0 Hz, H-3β), 3.87 (3H, s, OMe-4′), 5.51–5.40 (1H, m, H-2), 5.96 (1H, dd, *J* = 2.2 Hz, *Jo* = 0.7 Hz, H-8), 5.98 (1H, dd, *J* = 2.23 Hz, *Jo* = 0.9 Hz, H-6), 6.99 (1H, d, *J* = 1.7 Hz, H-2′), 7.08–7.03 (2H, m, H-5′, H-6′), 7.73 (1H, s, OH-3′), 9.67 (1H, s, OH-7), 12.19 (1H, s, OH-5); ^13^C NMR (100 MHz, aseton-*d*_6_): 43.53 (C-3), 56.33 (OMe-4′), 79.82 (C-2), 95.88 (C-8), 96.83 (C-6), 103.23 (C-10), 112.32 (C-5′), 114.39 (C-2′), 118.78 (C-6′), 132.82 (C-1′), 147.61 (C-3′), 148.71 (C-4′), 164.29 (C-5), 165.29 (C-9), 167.42 (C-7), 197.14 (C-4);^43^ HRMS (ESI) (*m*/*z*) [M + H]^+^: 303.0863.

Metabolite 2 (M2) (eriodictyol) (3.6 mg) was isolated from the 50–52% *n*-hexane/ethyl acetate fraction of the same column chromatography. It was orange-pink at 365 nm on the TLC plate and has a more apolar structure than hesperidin.

^1^H NMR (400 MHz, DMSO-*d*_6_): 2.87–2.81 (1H m, H-3α), 3.55 (1H, d, *J* = 5.1 Hz, H-3β), 4.12 (1H, s, H-2), 5.34 (1H, s, H-6), 6.31(1H, s, H-8), 7.13 (2H, s, H-5′, H-6′), 7.30(1H, s, H-2′), 8.54 (1H, s, OH), 8.98 (1H, s, OH), 9.45 (1H, s, OH-7), 12.68 (1H, s, OH-5); ^13^C NMR (75 MHz, aseton-*d*_6_): 46.67 (C-3), 67.02 (C-2), 89.14 (C-8), 89.14 (C-6), 102.80 (C-10), 112.55 (C-2′), 113.51 (C-5′), 113.51 (C-6′), 131.73 (C-1′), 139.54 (C-3′), 143.70 (C-4′), 155.84 (C-5), 176.53 (C-9), 180.38 (C-7), 202.39 (C-4);^17,43^ HRMS (ESI) (*m*/*z*) [M + H]^+^: 289.1092.

### 4.5 *In Vitro* Antimicrobial Activity

#### 4.5.1 Microorganism Strains

*E. coli* NRRL B-3008, *S. aureus* ATCC 6538, *S. typhimurium* ATCC 13311, *Bacillus subtilis* NRRL B-4378, *S. boulardii* REFLOR ve *S. cerevisiae* ATCC 9763 and *C. albicans* ATCC 10231 and *A. nidulans* Abraham standard strains used in the antimicrobial experiments.

#### 4.5.2 Microdilution Assay

The broth microdilution assay method (CLSI, 2006) was used, and the samples were prepared in a 96-well microtiter format ranging from 500 to 15.6 g/mL.^44^ A bacterial suspension containing 1 × 10^7^ CFU/mL of the microorganism with a volume of 10 μL was added to each well. The broth with microorganisms in the last row was used as a negative control, whereas only the broth medium was used exclusively as a positive control. Twenty μL of resazurin was added to all wells after 24 h of incubation at 37 °C to stain the viable bacteria; however, 20 μL of resazurin was added to the fungi after 48 h. The minimum inhibitory concentrations (MIC, μg/mL) were defined as the first colored well. Chloramphenicol and amoxicillin (Sigma-Aldrich, Germany) were used as antibacterials. In contrast, amphotericin B and ketoconazole (Sigma-Aldrich, Germany) (MIC range: 0.125 μg/mL-64 μg/mL) were used as standard antifungal agents for comparison, where all experiments were replicated in three independent assays.

#### 4.5.3 Combination

The test samples were evaluated using the Checkerboard microdilution assay in the 96-well format as previously described.^45^ For this purpose, 2-fold dilutions of hesperetin and naringenin (128–0.25 μg/mL) were prepared for eight serial dilutions. 25 μL aliquots of sample were added to the wells in a vertical orientation, and 25 μL aliquots of each antibiotic’s dilution were added in a horizontal orientation so that the plate contained defined concentration combinations of the two compounds. Following this, each well was inoculated with a 50 μL (5 × 10^3^ CFU/well) turbidometrically standardized microorganism suspension and further incubated at 35 °C for 24 h. After incubation, 20 μL of resazurin was added to all wells and left at 35 °C for 2 h. The checkerboard method was performed using the fractional inhibitory concentration index (∑FICI), which was defined as the sum of the MIC of each sample when used in combination divided by the MIC of the sample when used alone.

Consequently, the types of effects were classified as follows: ∑FIC ≤ 0.5 = synergism; ∑FIC ≥ 0.5 and ≤1 = additive effect; ∑FIC > 1 and <4 = indifferent effect; ∑FIC ≥ 4 = antagonism.^46,47^

#### 4.5.4 Biofilm Assay

For screening hesperidin and its metabolites for antibiofilm potential, the biofilm activity method of Mariscal et al. (2009) was modified.^48^ For this purpose, *S. aureus* ATCC 6538 and *P. aeruginosa* ATCC 27853 pathogens were inoculated and developed at 37 °C for 18–24 h in tryptic soy broth (TSB). Then, biofilms were produced by pipetting 100 μL of inoculum into 96-well plates containing 5 mL of TSB. After mixing, the plates were incubated for a further 48 h at 37 °C. All wells containing biofilm carriers were rinsed three times with a sterile 0.9% saline solution for biofilm fixing. The concentrations of compounds were prepared in the range of 500–15.6 g/mL and then added in 96-well microtiter plates. After 24 h of incubation at 37 °C, 20 μL of resazurin was added, and the minimum biofilm inhibition concentration (MBIC) was calculated.

#### 4.5.5 Synergistic Activity in the Biofilm Using the Checkerboard Method

Eight serial dilutions of hesperetin and naringenin were combined, which were prepared using the checkerboard method and transferred into sterile 96-well microplates. Eight wells in the same plate were prepared as growth and sterility control.

The work reported^49^ by Stanojevic et al. (2010) was modified and used. In this context, biofilm was prepared as described in the biofilm activity study with the *S. aureus* strain. After two washes, 8 horizontal rows of naringenin in the combination and 8 vertical rows of hesperetin were added to the plate at decreasing concentrations, 50 μL for each compound, and 100 μL of medium was added on top. The combination of two compounds at different concentrations was prepared in 64-well format and incubated at 37 °C for 24 h. At the end of the incubation, 20 μL of Resazurin solution was added, and incubation continued for another 3 h using the same conditions. At the end of the incubation, no growth was observed in the blue-colored wells. Calculation was made according to the fractional inhibition concentration index (FICI) as described above.

### 4.6 In Vitro Anti-inflammatory Activity

As previously, inhibition of lipoxygenase (1.13.11.12, type I–B, 7.9 Unit/mg) enzyme activity was measured spectrophotometrically on a special 96-well quartz plate (Baylac and Racine, 2003; Biltekin et al., 2023).^50,51^ For 10 min at 25 °C, 1.94 mL of potassium phosphate buffer (100 mM; pH: 8.80), 40 mL of test compounds, and 20 mL of lipoxygenase enzyme were incubated. Each well received 300 μL of this mixture. The reaction was then started by adding 7.5 μL of linoleic acid solution and measuring the change in absorbance at 234 nm for 10 min. The assays were repeated four times. As a positive control, nordihydroguaiaretic acid (NDGA) was used. Calculation of the % inhibition is shown below
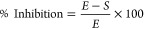
*E* the absorbance of the enzyme without a sample. *S* the absorbance of the enzyme with the test sample

### 4.7 Acute Toxicity Bioassay

The *Artemia* microwell assay was modified according to the Jacobsson method.^52^*Artemia* cysts (Artemio pur, JBL, Neuhofen, Germany) were hatched in a separation funnel containing artificial seawater (33 g of salt/L, Coral pro salt, Red Sea, Eilat, Israel) prepared in deionized water. A small aquarium air pump was used to aerate the water until the shrimp were harvested (24 h, RT). The samples were added in duplicates to flat-bottom 96-well plates *Artemia nauplii* (10–15) and were transferred to the wells and incubated at RT for 24 h. Toxicity was calculated as % dead or immobilized/total nauplii in each well.

### 4.8 Statistical Analysis

To compare differences in data between the standard and experimental groups, statistical analysis was done using GraphPad Prism Version 8.0. The results were expressed as the mean ± the standard deviation (S.D.). Statistically significant values were compared using two-way ANOVA with the Tukey Multiple Comparison Test, and *p*-values of less than 0.05 were considered statistically significant by IBM SPSS Statistics 22.

## Supporting Information Available

Mass spectra and NMR data of hesperidin and its metabolites (PDF)
