# Supplementary material for: Microbial Transformation of Hesperidin and Biological Evaluation
Source: ACS Omega. 2023 Nov 1;8(45):42610–21. doi: 10.1021/acsomega.3c05334 (PMC10652256; doi:10.1021/acsomega.3c05334)
Supplement: Supplementary file 1 — ao3c05334_si_001.pdf [file ao3c05334_si_001.pdf]

-SUPPORTING INFORMATION-

Microbial Transformation of Hesperidin and Biological Evaluation

Damla Kırcı[1*], Fatih Demirci[2,3], Betül Demirci[2*]

[1] Department of Pharmacognosy, Faculty of Pharmacy, Selçuk University, Konya, Türkiye

[2] Department of Pharmacognosy, Faculty of Pharmacy, Anadolu University, Eskişehir, Türkiye

[3] Faculty of Pharmacy, Eastern Mediterranean University, Famagusta, N. Cyprus, via Mersin Türkiye

*To whom correspondence should be addressed:
Dr. DAMLA KIRCI
Tel.: +90 539-799-6898
E-mail: damla.kirci@selcuk.edu.tr

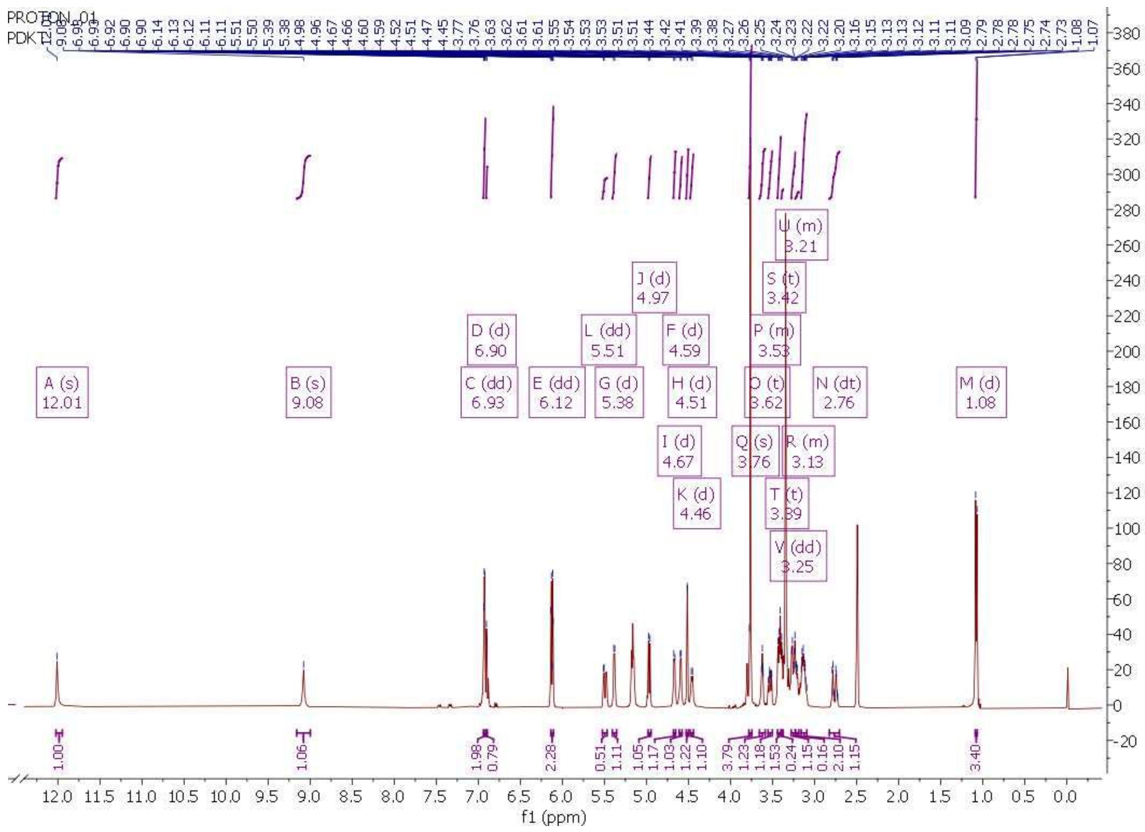

Figure S1. ^{1}H-NMR spectrum of hesperidin

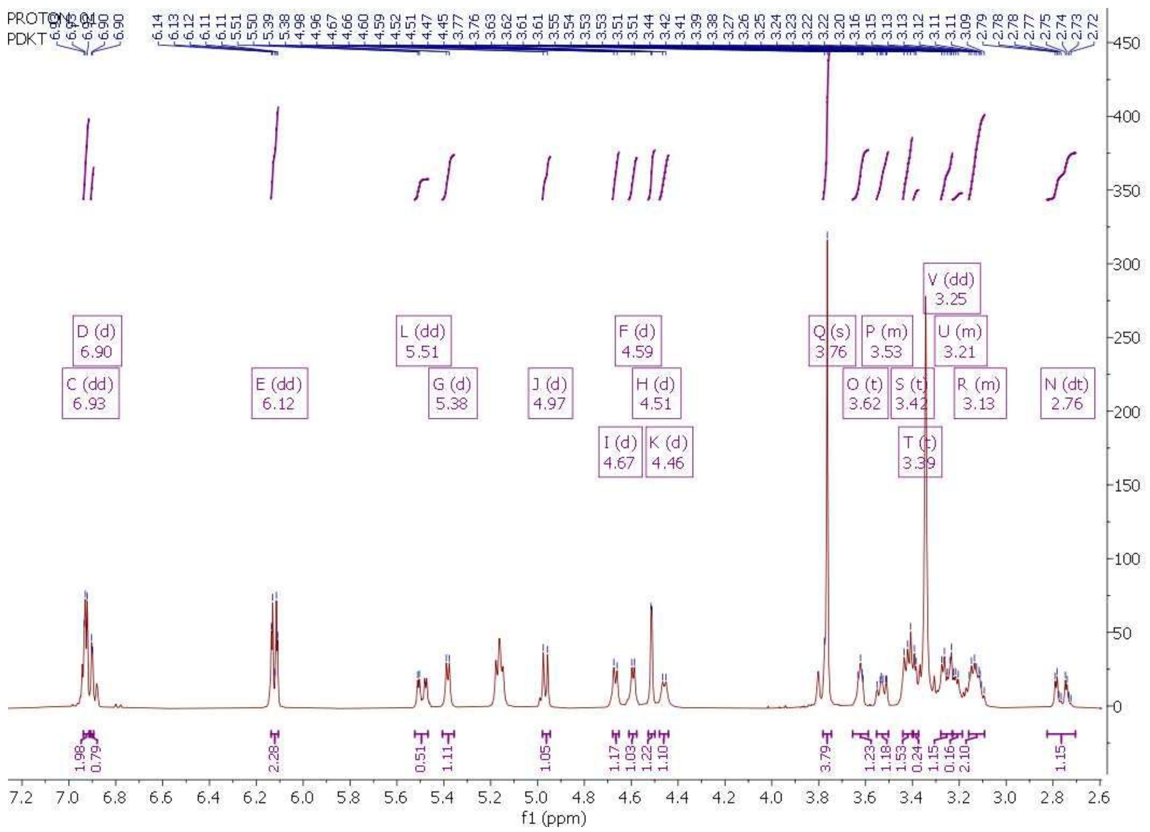

Figure S2. ^{1}H-NMR spectrum of Hesperidin (2.6-7.2 ppm)

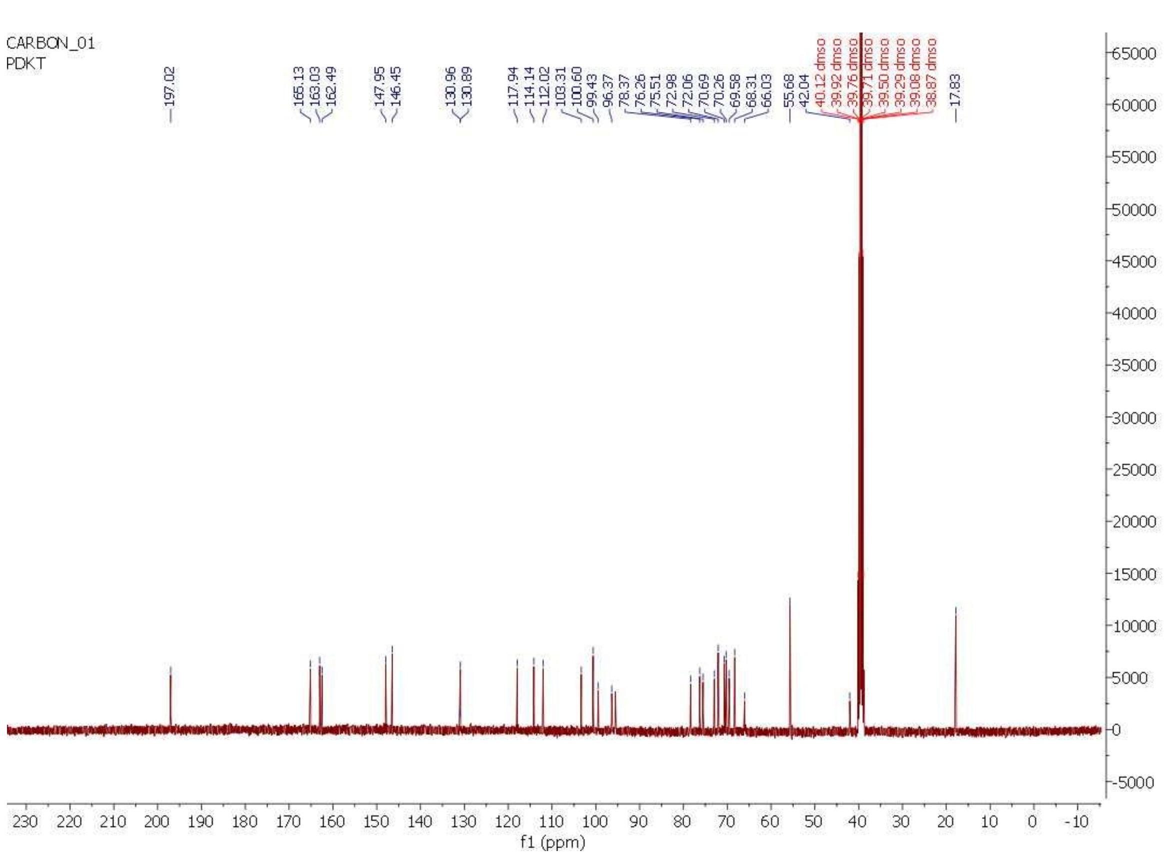

Figure S3. ^{13}C-NMR spectrum of hesperidin

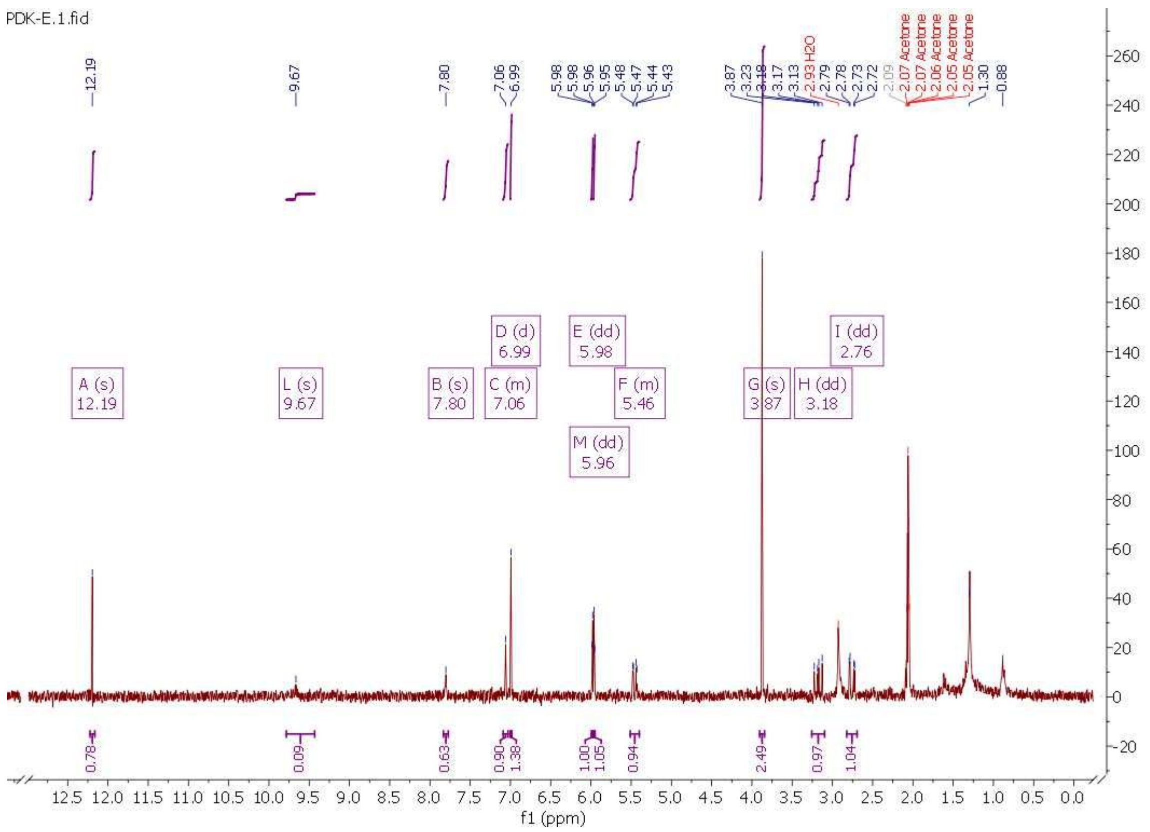

Figure S4. ^{1}H-NMR spectrum of hesperitin

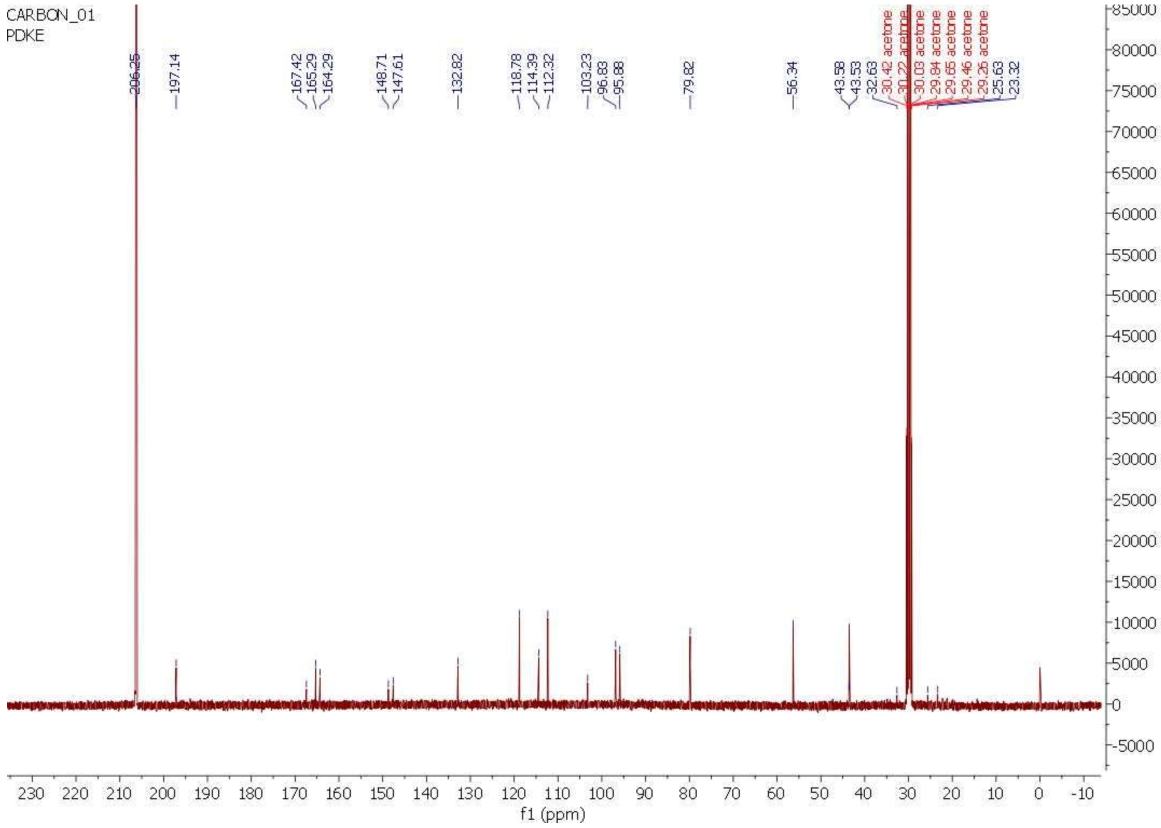

Figure S5. ^{13}C-NMR spectrum of hesperetin

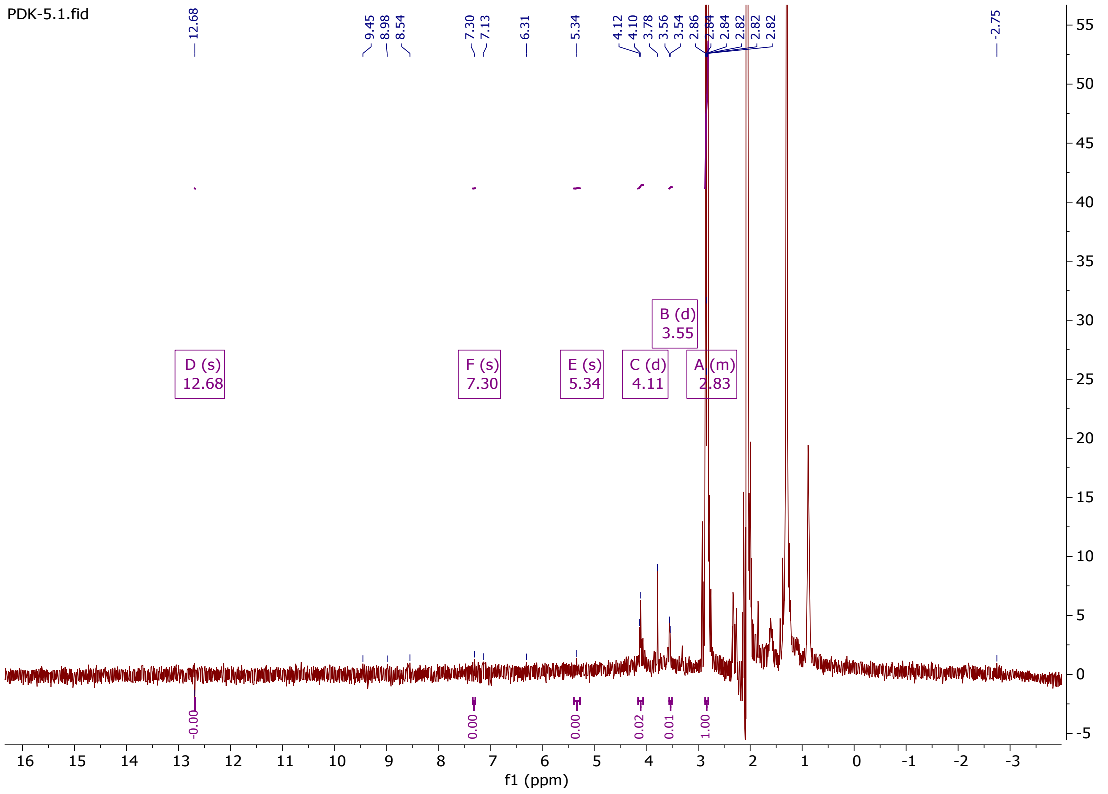

Figure 6. ^{1}H-NMR spectrum of eriodictyol

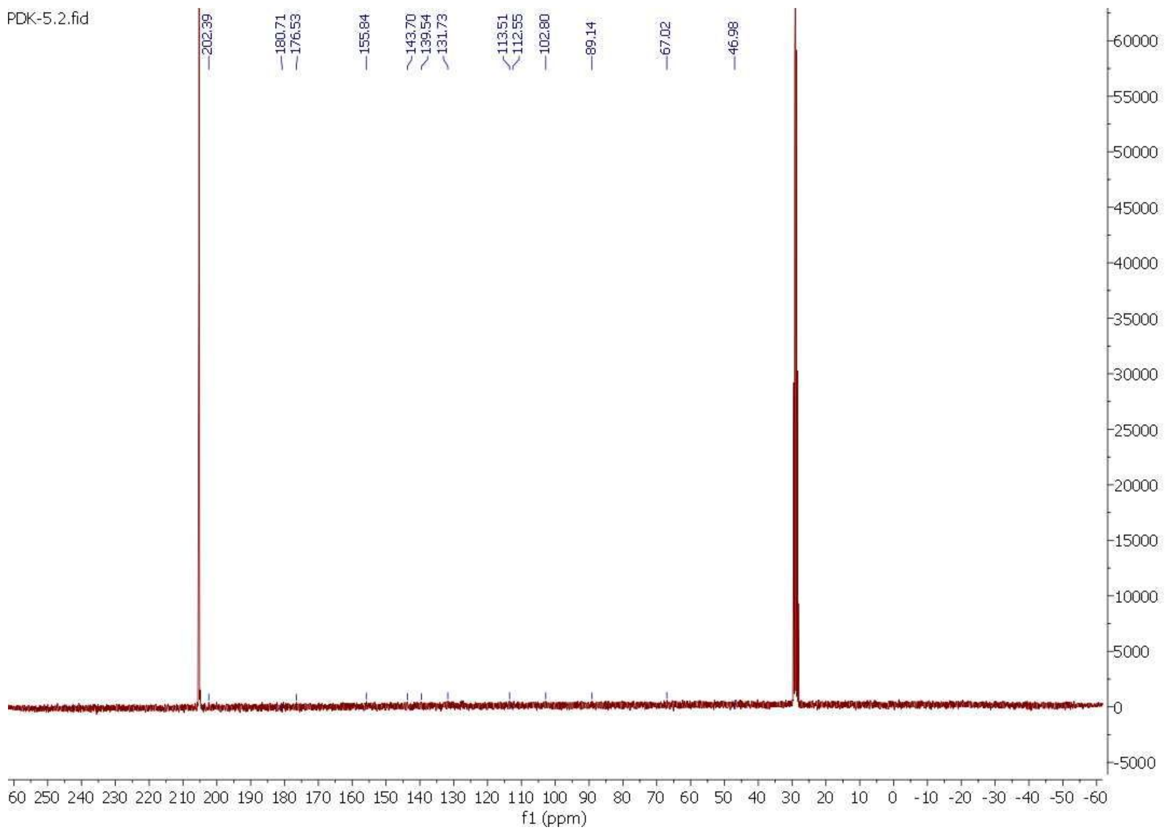

Figure 7. ^{13}C-NMR spectrum of eriodictyol
